# Decision curve analysis confirms higher clinical utility of multi-domain versus single-domain prediction models in patients with open abdomen treatment for peritonitis

**DOI:** 10.1186/s12911-023-02156-w

**Published:** 2023-04-06

**Authors:** Markus Huber, Patrick Schober, Sven Petersen, Markus M. Luedi

**Affiliations:** 1 Department of Anaesthesiology and Pain Medicine, Bern University Hospital, Inselspital, University of Bern, Freiburgstrasse 10, Bern, 3010 Switzerland; 2 Department of Anaesthesiology, Amsterdam University Medical Centres, Vrije Universiteit Amsterdam, Amsterdam, the Netherlands; 3 Department of General and Visceral Surgery, Asklepios Hospital Altona, Hamburg, Germany

**Keywords:** Peritonitis, Machine learning, Decision curve analysis, Clinical prediction modelling, Calibration

## Abstract

**Background:**

Prediction modelling increasingly becomes an important risk assessment tool in perioperative systems approaches, e.g. in complex patients with open abdomen treatment for peritonitis. In this population, combining predictors from multiple medical domains (i.e. demographical, physiological and surgical variables) outperforms the prediction capabilities of single-domain prediction models. However, the benefit of these prediction models for clinical decision-making remains to be investigated. We therefore examined the clinical utility of mortality prediction models in patients suffering from peritonitis with a decision curve analysis.

**Methods:**

In this secondary analysis of a large dataset, a traditional logistic regression approach, three machine learning methods and a stacked ensemble were employed to examine the predictive capability of demographic, physiological and surgical variables in predicting mortality under open abdomen treatment for peritonitis. Calibration was examined with calibration belts and predictive performance was assessed with the area both under the receiver operating characteristic curve (AUROC) and under the precision recall curve (AUPRC) and with the Brier Score. Clinical utility of the prediction models was examined by means of a decision curve analysis (DCA) within a treatment threshold range of interest of 0–30%, where threshold probabilities are traditionally defined as the minimum probability of disease at which further intervention would be warranted.

**Results:**

Machine learning methods supported available evidence of a higher prediction performance of a multi- versus single-domain prediction models. Interestingly, their prediction performance was similar to a logistic regression model. The DCA demonstrated that the overall net benefit is largest for a multi-domain prediction model and that this benefit is larger compared to the default “treat all” strategy only for treatment threshold probabilities above about 10%. Importantly, the net benefit for low threshold probabilities is dominated by physiological predictors: surgical and demographics predictors provide only secondary decision-analytic benefit.

**Conclusions:**

DCA provides a valuable tool to compare single-domain and multi-domain prediction models and demonstrates overall higher decision-analytic value of the latter. Importantly, DCA provides a means to clinically differentiate the risks associated with each of these domains in more depth than with traditional performance metrics and highlighted the importance of physiological predictors for conservative intervention strategies for low treatment thresholds. Further, machine learning methods did not add significant benefit either in prediction performance or decision-analytic utility compared to logistic regression in these data.

**Supplementary Information:**

The online version contains supplementary material available at 10.1186/s12911-023-02156-w.

## Introduction

Advanced statistical methods such as those summarized under the term machine learning are becoming ever more important in the analysis of medical data as illustrated by the rapid and exponential increase in the number of machine learning papers [1]. Such methods are increasingly used for clinical predictive modelling to quantify individual patients’ risks [2] and to allocate optimal resources. The central elements of these models are a set of predictors such as demographic variables, comorbidities, biomarkers and characteristics of clinical interventions, e.g. to predict the risk of hospital mortality for critically ill hospitalized adults with the Acute Physiology And Chronic Health Evaluation (APACHE) III prognostic system [3] and the Simplified Acute Physiology Score (SAPS III) [4]. Recent evidence demonstrates that combining predictors from multiple medical domains (demographics, physiological and surgical) features higher predictive performance metrics than using predictors only from a single domain, for example in patients with open abdomen treatment for peritonitis [5]. Despite decades of extensive research, prognosis of abdominal sepsis remained poor [6] and the potential key contributors to the pathology unknown [7].

However, several issues in terms of building, evaluating and using such clinical prediction models and questions regarding the added benefit of advanced machine learning methods over traditional statistical methods have been noted recently: First and in terms of *model evaluation*, the important issue of calibration has traditionally received only little attention relative to the issue of model performance [8]. Calibration refers to the level of agreement between observed risk and predicted risk, and prediction models with poor calibration can be misleading [9]. Modern methods such as the calibration belt were introduced to facilitate the computation and interpretation of the level of calibration of a prediction model [10].

Second and in terms of *model performance*, the discrimination capacity of these prediction models is traditionally evaluated by means of the area under the receiver operating characteristic curve (AUROC) [11]. Prediction modelling efforts are often used with imbalanced data where the adverse outcome occurs only rarely: While the AUROC is formally not biased towards such a minority class [12], the AUROC metric might suggest a too optimistic impression of the classifier in highly imbalanced data [13]. In such cases, the examination of the precision-recall curve and the associated area under the curve (AUPRC) provides more information [14]. In addition, when averaged across all probability thresholds, AUROC treats sensitivity and specificity as equally important and thus ignores clinical relevant information of the misclassification costs [15].

Third and in terms of *clinical utility*, it has been noted that the question how and if such prediction models can improve care delivery and disease management cannot be determined by solely inspecting model discrimination or model calibration [16, 17]. Introduced in the year 2006, Decision Curve Analysis (DCA) provides a novel way to address the clinical consequences of prediction models in terms of diagnostic and prognostic strategies beyond traditional evaluation metrics such as the AUROC [18]. DCA centres around the concept of net benefit (NB), which is a measure that explicitly incorporates weights for detecting true positives (with the disease) versus diagnosing false positives (without the disease) and is expressed in units of true positives [19]. Overall, DCA allows to evaluate whether a prediction model exhibit higher net benefits as the two default intervention strategies of “treat all” and “treat none” [20]. DCA has been employed in various medical domains such as radiology [21], oncology [22] and many other areas [23, 24].

Fourth and in terms of *machine learning for prediction modelling*, a systematic reviews showed no benefit in terms of performance gain by more advanced statistical machine learning algorithms over the traditional method of logistic regression [25]. To leverage the prediction capacity of various machine learning methods more generally, the concept of a stacked learner was introduced. A stacked learner is able to systematically combine the predictions of several so-called base learners [26]. Stacked learners were recently employed to predict mortality risk scores in an elderly population [27], the risk of 30-day readmission after bariatric surgery in the United States [28], the COVID-19 severity among patients with cardiovascular conditions [29] and the mortality in intensive care units [30].

In this study, we aim at applying these methods holistically to a clinical prediction model for a previously defined cohort of patients with open abdomen treatment for peritonitis, a clinically challenging cohort deserving optimal resource allocation at the time of admission to intensive care. While the performance benefit of using predictors from multiple medical domains could be recently demonstrated [5], the benefit of a multi-domain prediction approach in terms of clinical utility and decision making remains unknown and yet to be quantified. We adopt a broad, general perspective on the possible interventions based on the predicted mortality risks by the models, i.e. possible interventions could constitute running additional diagnostic tests and laboratory measurements or modifications of the existing treatment plan. Clinical preferences in terms of the performing the intervention are represented by the treatment threshold probability [31] and the particular choice of a reasonable threshold probability range should be the initial step of a decision curve analysis [32]. Given the severity of the primary outcome (mortality) in this study, we limit the analysis of the net benefit of the prediction models to the threshold probability range of 0–30%: that is, we are more much more concerned about the patients’ possible death than about unnecessary interventions for surviving patients.

The aim, therefore, is to examine if single- and multi-domain prediction models provide a higher net benefit than the default intervention strategy of “treat all” (where the intervention consists of the broad interventions mentioned above). In addition, we employ several base machine learners and a stacked ensemble to examine the potential benefit of modern machine learning methods compared to regular and penalized logistic regression both in terms of model performance and in terms of clinical utility by means of a decision curve analysis.

## Methods

### Clinical data

This is a secondary analysis of a study performed in adherence to the principles in the Declaration of Helsinki and which was approved by the Hamburg Medical Association (#WF072/20). In brief, the initial study described a cohort of 1,351 consecutive adult patients surgically treated for peritonitis over a decade (January 1998 to December 2018) at the Department of General and Visceral Surgery of the Asklepios Hospital Altona, Hamburg, Germany, with survival (died vs. survived during stay of hospital) as primary outcome measure [5]. The primary study further describes details regarding data collection, the standard surgical procedures and the definition of wound healing disorders. We note that the sample size of the primary study consisted of a convenience sample.

### Summary statistics

Categorical variables were summarized by counts and percentages and compared by means of a chi-square test. Numerical variables were summarized with median and interquartile range and compared by means of an unpaired two-sample Wilcoxon test.

### Prediction modelling

The study follows the network “Enhancing the QUAlity and Transparency Of health Research” (EQUATOR)’s guideline for transparent reporting of a multivariable prediction model for individual prognosis or diagnosis (TRIPOD) [33].

**Fig. 1:**
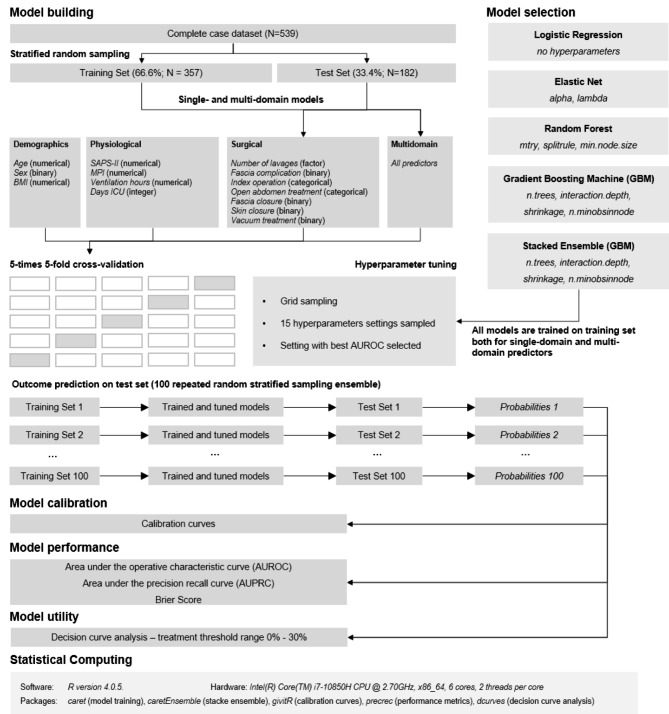
Model building and evaluation approach in this study. A detailed description of the approach is provided in the Methods section

As recommended in the literature [34], Fig. 1 illustrates the model building and evaluation approach of this study. We employed the following statistical methods to build a clinically prediction model of mortality: multivariable logistic regression, Elastic Net, Random Forest and Gradient Boosting Machine (GBM). Each of those methods was trained with predictors from single medical domains (“single-domain prediction models”) and with all available predictors (“multi-domain prediction model”). The particular medical domain of each predictor is provided in Table 1 and in Fig. 1. A stacked learner [26] was derived by combining the cross-validated predicted probabilities of the individual base learners by means of a Gradient Boosting Machine, thus resulting in stacked learner for each single-domain and the multi-domain case. A GBM as the so-called meta-learner was chosen to handle possible correlated predicted probabilities from the individual base models, however, we note that also other algorithms are possible choices for the stacked learner, i.e. a neural network or a generalized linear model.

**Table 1:** Patients’ characteristics stratified according to clinical outcome (survived versus died) [5]. Data availability is indicated for each variable

*Complete Case Analysis*	All patients	Survived	Died	*p*
	*N = 539 (100%)*	*N = 395 (73.3%)*	*N = 144 (26.7%)*	
*Demographics*				
**Sex** (female)	221 (41.0%)	161 (40.8%)	60 (41.7%)	0.93
**Age** (years)	70.0 [59.0;76.0]	67.0 [56.0;75.0]	73.0 [66.0;79.0]	< 0.001
**BMI** (kg.m^− 2^)	25.3 [22.9;28.8]	25.1 [22.8;28.2]	25.9 [23.0;29.4]	0.30
*Physiology*				
**SAPS-II Score**	46.0 [37.0;57.0]	42.0 [34.5;52.0]	56.5 [47.0;68.5]	< 0.001
**Mannheimer Peritonitis-Index** (MPI)	21.0 [14.0;28.5]	20.0 [13.0;26.0]	27.0 [16.0;33.0]	< 0.001
**Duration of ventilation** (hours)	144 [58.5;414]	138 [60.0;359]	223 [54.8;502]	0.031
**Days in ICU**	10.0 [3.00;21.0]	10.0 [4.00;20.5]	11.0 [2.00;22.2]	0.97
*Surgery*				
**Number of lavages**:				0.25
1	321 (59.6%)	245 (62.0%)	76 (52.8%)	
2	125 (23.2%)	83 (21.0%)	42 (29.2%)	
3	47 (8.72%)	36 (9.11%)	11 (7.64%)	
4	21 (3.90%)	15 (3.80%)	6 (4.17%)	
5	12 (2.23%)	7 (1.77%)	5 (3.47%)	
>5	13 (2.41%)	9 (2.28%)	4 (2.78%)	
**Wound healing disorder** (Yes)	131 (24.3%)	110 (27.8%)	21 (14.6%)	0.002
**Fascia complication** (Yes)	36 (6.68%)	27 (6.84%)	9 (6.25%)	0.96
**Index operation**:				0.75
Median	131 (24.3%)	93 (23.5%)	38 (26.4%)	
Transverse	342 (63.5%)	252 (63.8%)	90 (62.5%)	
Other	66 (12.2%)	50 (12.7%)	16 (11.1%)	
**Open abdomen treatment**:				0.92
Median	142 (26.3%)	105 (26.6%)	37 (25.7%)	
Transverse	383 (71.1%)	279 (70.6%)	104 (72.2%)	
Other	14 (2.60%)	11 (2.78%)	3 (2.08%)	
**Fascia closure** (Yes)	487 (90.4%)	391 (99.0%)	96 (66.7%)	< 0.001
**Skin closure** (Yes)	485 (90.0%)	389 (98.5%)	96 (66.7%)	< 0.001
**Vacuum treatment** (Yes)	42 (7.79%)	33 (8.35%)	9 (6.25%)	0.53

In terms of model training for each method, a repeated random stratified sampling with respect to the outcome variable was performed where 66% of the available data was assigned to a training set and the remaining 34% to a test set. Fifteen hyperparameter settings for each tunable hyperparameter were randomly sampled for each round of a 5-times repeated 5-fold cross-validation. The following hyperparameters were available for tuning: *alpha* (mixing parameter, with 0 ≤ alpha ≤ 1) and *lambda* (shrinkage parameter) for Elastic Net; *mtry* (number of variables to possibly split at in each node), *splitrule* (splitting rule), *min.node.size* (value of minimal node size) for Random Forest and *n.trees* (total number of trees to fit), *interaction.depth* (maximum depth of each tree), *shrinkage* (shrinkage parameter) and *n.minobsinnode* (minimum number of observations in the terminal nodes of the trees) for Gradient Boosting Machine. The hyperparameters with the largest AUROC value were chosen as the optimal hyperparameters.

Calibration of the prediction models was assessed by means of calibration belts [10] and associated p-values of a calibration test which examines the departure from perfect calibration [35]: a low p-value (p < 0.05) refers to a miscalibrated prediction model and perfect calibration is represented by the diagonal line in the calibration plot. Predictive performance was evaluated by the area under the receiver operating characteristic curve (AUROC) and the area under the precision recall curve (AUPRC). We further computed the Brier Score as performance metric [36]: The Brier score of a model accounts for both calibration and utility of the predictions by the model and lower scores are considered better [37]. The model training and evaluation process was repeated 100 times to derive calibration and performance estimates on 100 test sets (Fig. 1), thus providing a computationally extensive and robust internal evaluation. P-values from the calibration test as well as performance metrics are summarized by the median value and associated 95%-confidence interval.

### Decision curve analysis

Decision curves are computed based on the model predicted probabilities for the test dataset of each random split-sample, allowing to illustrate both the median and 95%-confidence interval of the net benefit for a given treatment threshold. The decision curves were computed for each base learner and the stacked learner and separately for each medical domain within the treatment threshold range of interest (0-30%). To aid the interpretation of the decision curve, we note that the maximum net benefit is equal to the mortality prevalence [38, 39], which is 26.7% in the complete-case analysis.

### Missing data and statistical software

The original dataset contained several systematically missing data not at random [40] as discussed in the primary publication [5]. We therefore conducted two analysis: the primary analysis is based on a complete-case analysis. A full sensitivity analysis based on the original dataset and featuring a single imputation approach is presented in the Supplementary Material. In the single imputation approach, missing values for binary or categorical predictors are imputed by the mode and numerical variables are imputed by means of the median value. Importantly, the imputation is performed for each split-sample. Thus, the combination of a large ensemble of randomly drawn split-samples and split-sample specific imputation approach allows for a robust uncertainty quantification (Supplementary Figure SM1).

In terms of statistical software, the prediction models were computed with the *caret* package [41] decision curves were calculated with the *dcurves* package [42]. All computations were performed with R version 4.0.5 [43].

## Results

### Patients’ characteristics

Patients’ characteristics are summarized in Table 1 and shows that 395 patients (73.3%, 95%-CI: 69.3 − 77.0%) survived and 144 patients (26.7%, 95%-CI: 23.0 − 30.7%) died. Demographic predictors include gender, age and body mass index (BMI), whereas physiological predictors feature the SAPS-II score, the Mannheimer Peritonitis (MPI) Index, duration of ventilation and hemofiltration and days in the ICU. Predictors related to the surgical intervention include number of lavages, wound healing disorder, fascia complication, the type of index operation and open abdomen treatment, the use of vacuum treatment as well as the fascia and skin closure.

### Calibration

**Fig. 2:**
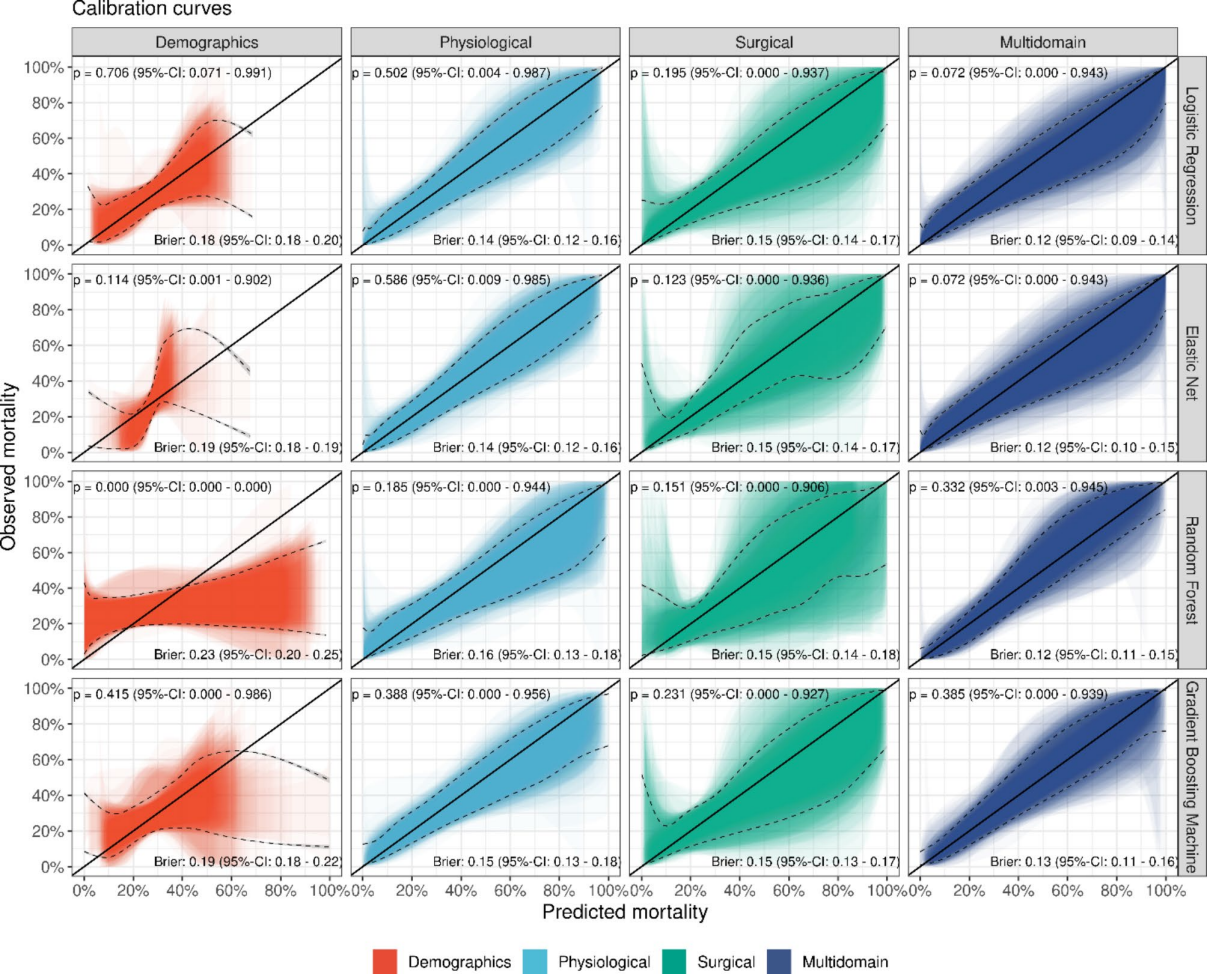
Calibration curves for the single-domain prediction models and multi-domain predictions models stratified according to the modelling approach. Shaded areas denote the 95%-confidence intervals. P-values regarding the quality of the calibration [10] and Brier-scores are shown for each prediction model and are summarized by the mean and 95%-confidence intervals. Black dashed lines indicate the Generalized Additive Model (GAM)-smoothed calibration belts from the ensemble of 100 individual calibration belts

The calibration in both single- and multi-domain models for different types of statistical approaches is shown in Fig. 2. In total, 100 calibration belts are shown for each prediction models: that is, one calibration belt for each of the 100 random training-test-splits (Fig. 1). Overall, the prediction models show a tendency to overestimate the mortality risk for medium to high risks (e.g. predicted probabilities in the range of around 50-100%), notably for surgical predictors. In terms of the particular underlying algorithm of the prediction model, the calibration properties of the physiological and surgical prediction models are similar. For demographic predictors, however, the calibration strongly depends on the underlying statistical approach. For the multi-domain prediction models, more advanced machine learning models like the Gradient Boosting Machine show slightly better calibration than regular and penalized logistic regression models, where the latter models tend to overestimate the mortality risk for medium to high risks.

Similar to the primary study which only used logistic regression, Fig. 1 highlights that the multi-domain prediction models generally demonstrate better calibration, however, some degree of miscalibration remains. Figure 2 highlights that the Brier score improved from single domain prediction models (median Brier scores of 0.14–0.23) to multidomain prediction models (median Brier scores of 0.12–0.13) similarly for the four statistical methods.

### Performance Metrics

An overview of the performance metrics of the various prediction models is shown in Table 2. There are two main findings in terms of predictive performance. First and similar to the primary study, the discrimination capacity is lowest for demographic predictors (median AUROC of 0.55–0.64) and highest for the multi-domain models (median AUROC of 0.84–0.86). Second, the predictive performance of more advanced machine learning methods is almost the same as with logistic regression, despite systematic tuning of hyperparameters.

**Table 2:** Performance metrics for the four base learners and the stacked ensemble. Median and 95%-confidence intervals are shown for the predictions in the test set in random repeated subsampling framework (see Methods)

Method	Domain	AUROC	AUPRC	Brier Score
**Logistic Regression**	Demographics	0.64 (95%-CI: 0.58–0.71)	0.39 (95%-CI: 0.32–0.48)	0.19 (95%-CI: 0.18–0.20)
	Physiological	0.81 (95%-CI: 0.75–0.86)	0.66 (95%-CI: 0.57–0.75)	0.14 (95%-CI: 0.12–0.16)
	Surgical	0.70 (95%-CI: 0.63–0.76)	0.60 (95%-CI: 0.50–0.67)	0.15 (95%-CI: 0.14–0.18)
	Multidomain	0.87 (95%-CI: 0.80–0.91)	0.75 (95%-CI: 0.65–0.84)	0.12 (95%-CI: 0.09–0.14)
**Elastic Net**	Demographics	0.65 (95%-CI: 0.58–0.72)	0.40 (95%-CI: 0.31–0.49)	0.19 (95%-CI: 0.18–0.19)
	Physiological	0.81 (95%-CI: 0.76–0.86)	0.66 (95%-CI: 0.57–0.75)	0.14 (95%-CI: 0.12–0.16)
	Surgical	0.70 (95%-CI: 0.63–0.76)	0.59 (95%-CI: 0.49–0.67)	0.15 (95%-CI: 0.14–0.17)
	Multidomain	0.86 (95%-CI: 0.81–0.90)	0.74 (95%-CI: 0.63–0.81)	0.12 (95%-CI: 0.10–0.15)
**Random Forest**	Demographics	0.55 (95%-CI: 0.50–0.61)	0.30 (95%-CI: 0.26–0.38)	0.23 (95%-CI: 0.20–0.25)
	Physiological	0.77 (95%-CI: 0.71–0.83)	0.60 (95%-CI: 0.50–0.70)	0.16 (95%-CI: 0.13–0.18)
	Surgical	0.68 (95%-CI: 0.62–0.75)	0.57 (95%-CI: 0.49–0.67)	0.16 (95%-CI: 0.14–0.18)
	Multidomain	0.85 (95%-CI: 0.79–0.89)	0.72 (95%-CI: 0.64–0.80)	0.12 (95%-CI: 0.11–0.15)
**Gradient Boosting Machine**	Demographics	0.61 (95%-CI: 0.53–0.68)	0.33 (95%-CI: 0.28–0.40)	0.19 (95%-CI: 0.18–0.22)
	Physiological	0.79 (95%-CI: 0.73–0.85)	0.60 (95%-CI: 0.49–0.73)	0.15 (95%-CI: 0.13–0.18)
	Surgical	0.70 (95%-CI: 0.65–0.77)	0.60 (95%-CI: 0.51–0.68)	0.15 (95%-CI: 0.13–0.17)
	Multidomain	0.85 (95%-CI: 0.79–0.89)	0.71 (95%-CI: 0.60–0.80)	0.13 (95%-CI: 0.11–0.16)
**Stacked Ensemble**	Demographics	0.58 (95%-CI: 0.51–0.64)	0.32 (95%-CI: 0.26–0.40)	0.21 (95%-CI: 0.19–0.24)
	Physiological	0.80 (95%-CI: 0.72–0.85)	0.62 (95%-CI: 0.54–0.72)	0.15 (95%-CI: 0.13–0.18)
	Surgical	0.70 (95%-CI: 0.64–0.77)	0.58 (95%-CI: 0.48–0.66)	0.15 (95%-CI: 0.14–0.18)
	Multidomain	0.86 (95%-CI: 0.80–0.90)	0.74 (95%-CI: 0.63–0.82)	0.12 (95%-CI: 0.10–0.16)

AUROC: Area Under the Receiver Operating Characteristic Curve

AUPRC: Area Under the Precision Recall Curve

### Decision curve analysis

**Fig. 3:**
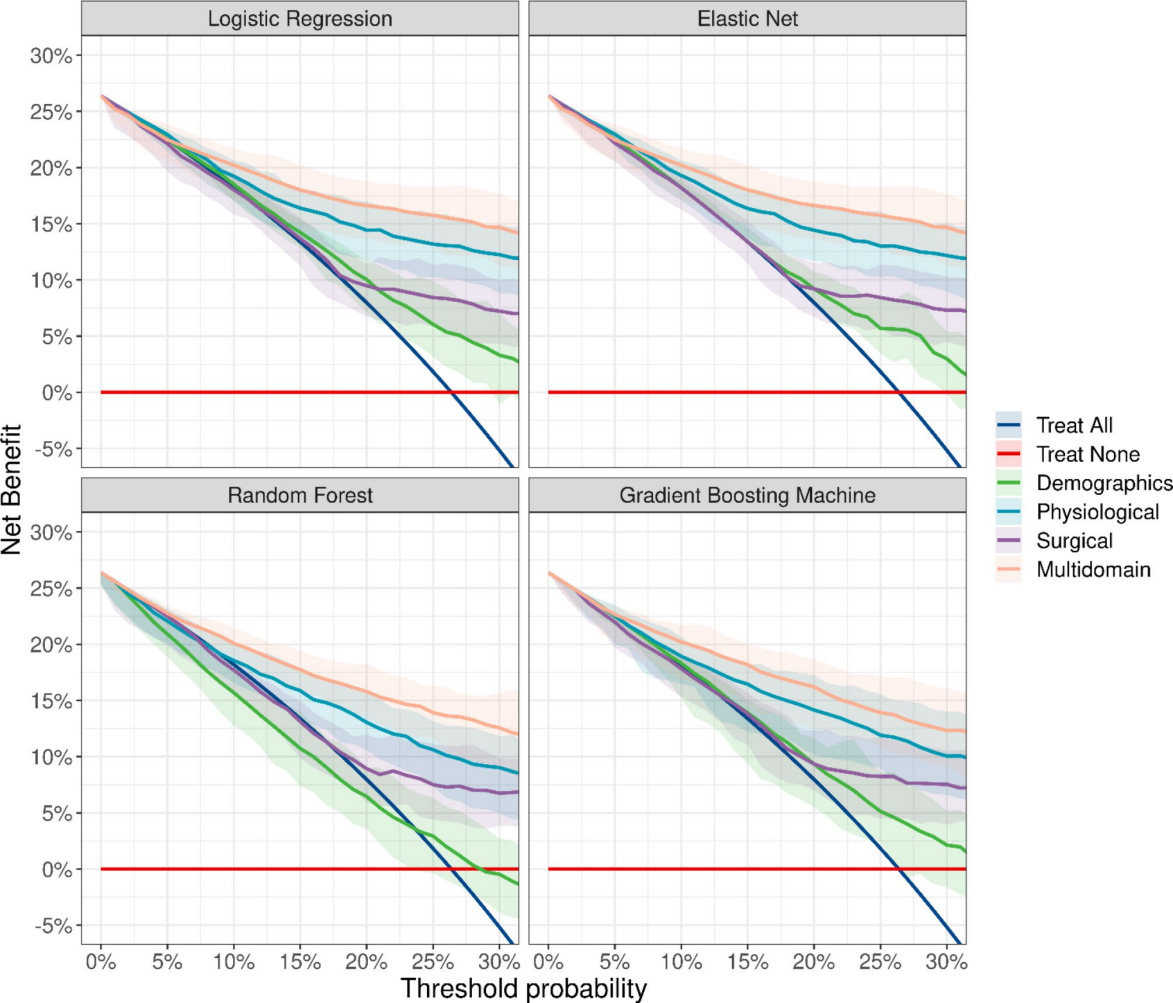
Decision curve analysis of single-domain and multi-domain models. The four domain-specific prediction models are compared to the two default strategies “Treat All” and “Treat None”. Note that the “Treat All” option crosses the zero benefit line at the prevalence of negative outcomes in our cohort and complete-case analysis (26.7%)

Figure 3 illustrates the decision curves for the single- and multi-domain prediction models for each statistical method separately for the threshold range of interest (0–30%). Overall, the four statistical methods considered here demonstrate that the multi-domain prediction offers the greatest decision-related benefit over the clinically relevant threshold probabilities with respect to the default intervention strategy of “treat all”. Importantly, the four methods agree in their quantitative estimate of the net benefit of a multi-domain prediction model (orange lines in Fig. 3).

Another result is that the decision-related net benefit of the prediction models is limited to threshold probabilities above about 10%: for lower thresholds, the net benefit is equal to the default intervention strategy of “treat all” and thus provides no added decision-related value. For threshold ranges between 10 and 20%, the decision-related added benefit from the prediction models derives predominately from physiological factors (blue lines in Fig. 3), which constitutes the most important domain in term of providing additional decision-related information. Surgical predictors provide only added net benefit compared to the default “treat all” strategy for threshold ranges above 20% and are secondary in terms of their benefit compared to physiological predictors. Prediction models based on demographic variables provide only marginal net benefits and are show dependency on the underlying statistical model: For example, a demographic prediction model based on a random forest demonstrates even smaller net benefits than the default “treat all” strategy.

### Stacked ensemble

We conclude by examining the calibration and decision-related characteristics of a stacked learner, which combines the prediction of the four individual base learners (logistic regression, Elastic Net, Random Forest and Gradient Boosting Machine). As the stacked learner is based on the cross-validated predicted probabilities of the individual base learners, it shares many calibration features of the individual base learners: For example, the stacked demographic prediction model features the same miscalibration as the demographic prediction model based on the random forest. However, the multi-domain prediction model of the stacked learner is well calibrated (p = 0.24). The performance metrics of the stacked ensemble are similar to the individual base learners (Fig. 3B). Additionally, the decision curves of the stacked learner lead to a similar interpretations as with the base learners (Fig. 3C).

## Discussion

**Fig. 4:**
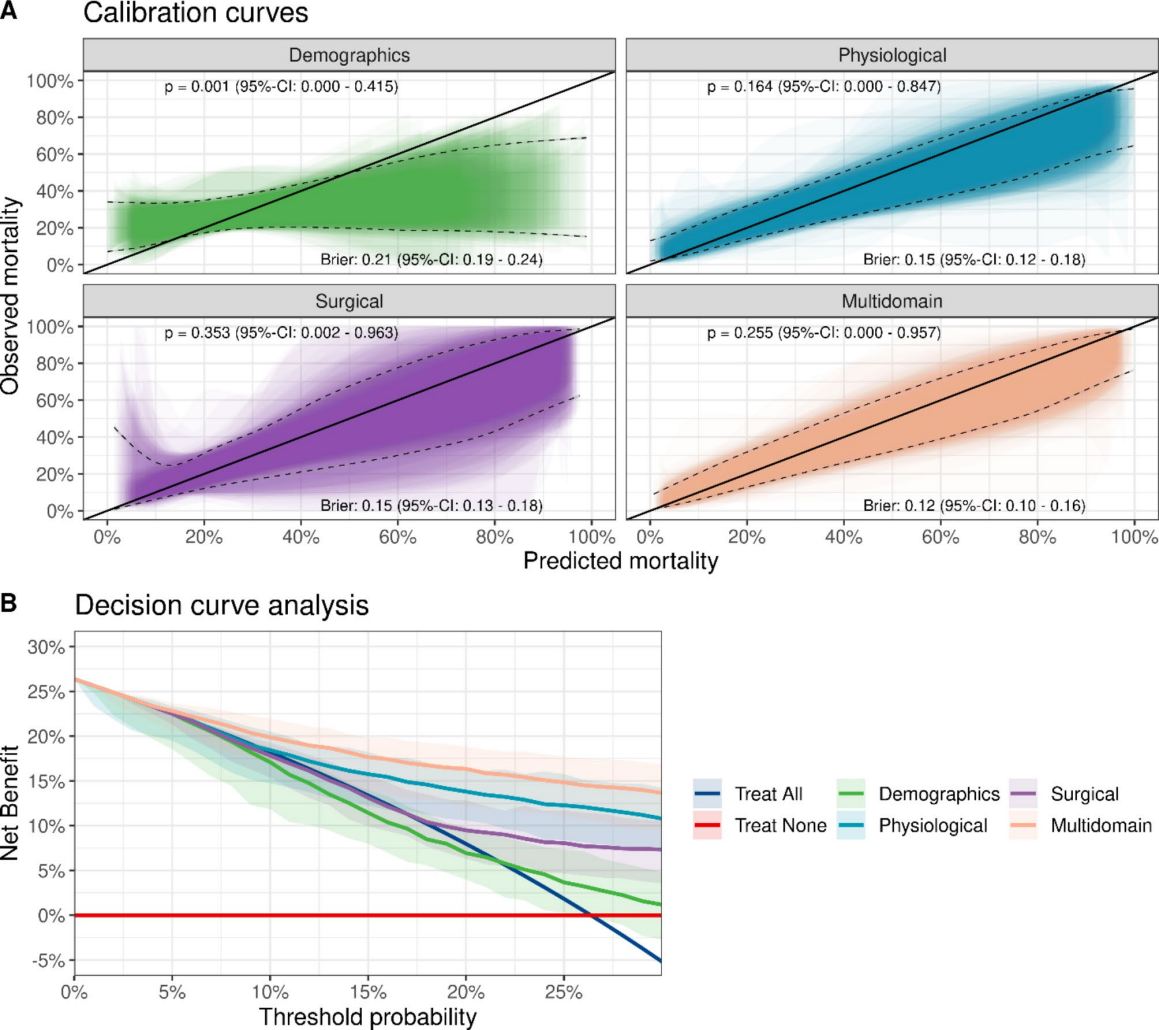
Calibration (A) and decision curve analysis (B) for a stacked ensemble prediction model based on a multivariable logistic regression, an Elastic Net, a Random Forest and a Gradient Boosting Machine as base learners. The stacked ensemble is based on a Gradient Boosting Machine that predicts the mortality outcome based on the cross-validated predictions of the base learners. For calibration, shaded areas denote the 95%-confidence range and black dashed lines indicate the Generalized Additive Model (GAM)-smoothed calibration belts from the ensemble of 100 individual calibration belts. P-values regarding the quality of the calibration and Brier-scores are shown

We assessed the benefit of machine learning methods both in the terms of predictive performance and clinical decision-related benefit in a large cohort of patients with open abdomen treatment for peritonitis. Similar to the primary analysis [5], the multi-domain prediction model based on more advanced statistical methods outperformed single-domain models in terms of discrimination capacity, however, individual machine learning methods (including the stacked ensemble) showed similar overall performance metrics (Table 2) and similar characteristics related to clinical decisions (Figs. 3 and 4).

The fact that more advanced machine learning prediction models demonstrate similar performance metrics as regular and penalized logistic regression is likely related to the sample size and the limited number of predictors: in these cases (“large N, small p”), a simulation study recently demonstrated that conventional logistic regression features good prediction performance compared to machine learning methods [44]. The fact that the stacked ensemble feature does not feature a significantly performance and clinical decision-related benefit is likely related to the tuning of the models hyperparameters towards optimal AUROC values, which results in very similar performing prediction models, whereas stacking generally benefits from diverse base learners [45]. We note, however, that there are theoretical und practical reasons for computing a stacked learner [46], for example to minimize the risk of choosing a particular prediction model that performs well on internal validation but may perform poorly on external validation and future data.

Against the current questions regarding the added benefit of machine learning methods in clinical prediction modelling for binary outcomes across medical domains [25] or in particular medical domains such as predicting heart failure events [47], we thus argue that considering all aspects from model building – including to the use of decision curve analysis as proposed recently [48] – might reveal further benefits of machine learning methods in outcome prediction. If these benefits might not be found in performance metrics and clinical utility, they might be found in other areas such as calibration for a stacked ensemble [30].

In terms of clinical importance, our decision curve analyses reveal different net benefits in terms of modelling clinical consequences in a suite of prediction models that are based on predictors from individual medical domains. Importantly, the framework of this study allows to assess the degree of risk aversion, for example in conservative risk-avoid strategy, for different medical domains and thus provides a much more nuanced perspective on risk modelling of patient with open abdomen treatment for peritonitis than the examination of traditional performance metrics. We focused on low threshold probabilities in the clinically-motivated range between 0 and 30% representing a conservative risk approach as we are more much more concerned about the patients’ possible death than about unnecessary interventions for surviving patients. We adopted a broad, general perspective on the possible interventions based on the predicted mortality risks by the models, i.e. possible interventions could constitute running additional diagnostic tests and laboratory measurements or modifications of the existing treatment plan.

Of clinical interest is that the decision curve framework revealed that the decision-related benefit of the prediction models is limited to threshold probabilities above around 10%: for lower threshold probabilities, the models’ benefit is equal to the default “treat all” intervention strategy. A further clinically important finding is that physiological predictors represent the domain with the largest decision-related benefit in the threshold range of interest of this study. The surgical predictors offer only secondary benefits which are limited to threshold ranges above 20%. Demographic predictors offer only marginal benefit. Importantly, these results are robust in terms of choice of the type of prediction model (i.e. logistic regression or a more advanced machine learning method).

Since this study demonstrated that different domain-specific prediction model differ (i) in the magnitude of net benefit and (ii) in the threshold range when they actually do provide a benefit (i.e. physiological predictor provide a decision-related benefit for lower threshold probabilities than surgical predictors), we conclude that the decision curve analysis framework provides a valuable tool to compare single-domain and multi-domain prediction models. While outcome prediction traditionally relies on clinical experience and single scores, these dimensions remain very basic and more sophisticated approaches such as the one described here are warranted. Additionally, a clinically-relevant consequence of the result that that the routinely collected variables that were available for this cohort of patients with open abdomen treatment for peritonitis did not provide additional decision-related benefit for threshold probabilities below 10% is that variables from further domains (i.e. laboratory measurements) should be examined for their benefit in this low treatment threshold range.

This study features inherent limitations. First, the dataset features a large degree of missing data not at random. A sensitivity analysis in the Supplementary Material using a single imputation approach to the missing data confirmed the conclusions from the complete case analysis. Second, the performance and decision-related aspects of the prediction models was evaluated only by means of internal validation and not by external validation, which is essential in terms of generalizing our results beyond the cohort of this secondary analysis [49]. Third, only a small sample of possible machine learning methods were employed as the main motivation of this study was to provide a holistic perspective on calibration, performance and clinical utility of predictors of different clinical domains. Additionally, the sampling of hyperparameter uncertainty was limited to those supported by the software library. Fourth, the prediction effort was limited to the available predictors and additional predictors might have resulted in a more detailed discussion regarding the benefit of machine learning methods in terms of discrimination capacity and clinical utility. In this context, a main limitation of this secondary study is that the sample size of the primary study consisted of a convenience sample and was not specifically powered to develop a clinical prediction model [50]. However, the main focus of this study was in examining the potential benefit of different clinical domains in terms of their clinical decision-related benefit rather than on the development of a state-of-the-art clinical prediction model for patients with open abdomen treatment for peritonitis, which would have required a much larger sample size and additional predictors. Thus, we argue that the available data – despite being limited – served the main purpose of the study. Fifth, the grouping of the variables into the physiological and surgical domain – whilst clinically motivated – could be done differently.

Overall, we emphasize that the results and conclusions stated above apply to the clinical prediction setting of this cohort of patients with open abdomen treatment for peritonitis and the chosen threshold range (0–30%). Depending of the clinical setting, different threshold ranges may be required which might result in different interpretations regarding the decision-related benefit of single-domain and multi-domain prediction models.

## Conclusions

In summary, in this cohort of patients with open abdomen treatment for peritonitis we found that machine learning methods did not add significant benefit either in prediction performance or decision-analytic utility compared to logistic regression within a treatment threshold range of interest of 0–30%. The decision curve analysis framework provides an invaluable tool to compare single-domain and multi-domain prediction model and demonstrates overall higher decision-analytic value of the latter and highlights different net benefits for demographic, physiological and surgical predictors of as a function of threshold probability. Thus, DCA provides the means to clinically differentiate the risks associated with each of these domains in more depth than with traditional performance metrics.

## Electronic supplementary material

Below is the link to the electronic supplementary material.

Supplementary Material 1

Supplementary Material 2

## Data Availability

The datasets used and/or analysed during the current study are available from the corresponding author on reasonable request.
