# Supplementary material for: Decision curve analysis confirms higher clinical utility of multi-domain versus single-domain prediction models in patients with open abdomen treatment for peritonitis
Source: BMC Med Inform Decis Mak. 2023 Apr 6;23:63. doi: 10.1186/s12911-023-02156-w (PMC10078078; doi:10.1186/s12911-023-02156-w)
Supplement: Supplementary file 2 — Supplementary Material 2 [file 12911_2023_2156_MOESM2_ESM.docx]

**SUPPLEMENTARY MATERIAL**

**Decision curve analysis confirms higher clinical utility of multi-domain versus single-domain prediction models in patients with open abdomen treatment for peritonitis**

Markus Huber^1^, Patrick Schober^2^, Sven Petersen^3^, Markus M. Luedi^1^

^1^ Department of Anaesthesiology and Pain Medicine, Inselspital, Bern University Hospital, University of Bern, Freiburgstrasse, 3010, Bern, Switzerland.

^2^ Department of Anaesthesiology, Amsterdam University Medical Centres, Vrije Universiteit Amsterdam, Amsterdam, the Netherlands

^3^ Department of General and Visceral Surgery, Asklepios Hospital Altona, Hamburg, Germany

*Corresponding author: Markus Huber, Dr. sc. ETH, Department of Anaesthesiology and Pain Medicine, Inselspital, Bern University Hospital, University of Bern, Bern, Switzerland, Freiburgstrasse 10, 3010 Bern, Switzerland, Tel: +41 31 664 12 15, Email: [markus.huber@insel.ch](mailto:markus.huber@insel.ch)

**Supplementary Tables**

|  | **All patients** | **Survived** | **Died** | ***p*** | **N** |
| --- | --- | --- | --- | --- | --- |
|  | ***N=1’351 (100%)*** | ***N=1’082 (80.1%)*** | ***N=269 (19.9)*** |  |  |
| *Demographics* |  |  |  |  |  |
| **Sex** (female) | 597 (44.3%) | 492 (45.6%) | 105 (39.2%) | 0.068 | 1’347 |
| **Age** (years) | 66.0 [54.0;75.0] | 64.0 [52.0;73.0] | 73.0 [63.0;78.0] | <0.001 | 1’351 |
| **Body Mass Index** (BMI; kg.m^-2^) | 25.1 [22.6;28.7] | 25.1 [22.7;28.4] | 25.5 [22.5;29.3] | 0.694 | 1’112 |
| *Physiology* |  |  |  |  |  |
| **Simplified Acute Physiology Score II** (SAPS-II) | 43.0 [34.0;54.0] | 40.0 [33.0;50.0] | 56.0 [45.0;66.0] | <0.001 | 1’323 |
| **Mannheimer Peritonitis-Index** (MPI) | 21.0 [14.0;28.0] | 19.0 [12.2;26.0] | 26.0 [16.0;32.2] | <0.001 | 598 |
| **Duration of ventilation** (hours) | 157 [64.0;402] | 140 [63.0;352] | 306 [74.0;538] | <0.001 | 1’309 |

| **ICU stay** (days) | 10.0 [4.00;21.0] | 9.00 [4.00;20.0] | 13.0 [3.00;24.0] | 0.019 | 1’351 |
| --- | --- | --- | --- | --- | --- |

| *Surgery* |  |  |  |  |  |
| --- | --- | --- | --- | --- | --- |
| **Number of lavages:** |  |  |  | 0.727 | 1’351 |
| 1 | 709 (52.5%) | 576 (53.2%) | 133 (49.4%) |  |  |
| 2 | 270 (20.0%) | 209 (19.3%) | 61 (22.7%) |  |  |
| 3 | 142 (10.5%) | 111 (10.3%) | 31 (11.5%) |  |  |
| 4 | 85 (6.29%) | 71 (6.56%) | 14 (5.20%) |  |  |
| 5 | 38 (2.81%) | 30 (2.77%) | 8 (2.97%) |  |  |
| >5 | 107 (7.92%) | 85 (7.86%) | 22 (8.18%) |  |  |
| **Wound healing disorder** (Yes) | 144 (24.0%) | 120 (27.4%) | 24 (14.9%) | 0.002 | 599 |
| **Fascia complication** (Yes) | 41 (6.84%) | 29 (6.62%) | 12 (7.45%) | 0.861 | 599 |
| **Index operation:** |  |  |  | 0.768 | 592 |
| Median | 146 (24.7%) | 104 (23.9%) | 42 (26.8%) |  |  |
| Transverse | 374 (63.2%) | 277 (63.7%) | 97 (61.8%) |  |  |
| Other | 72 (12.2%) | 54 (12.4%) | 18 (11.5%) |  |  |
| **Open abdomen treatment:** |  |  |  | 0.906 | 589 |
| Median | 158 (26.8%) | 117 (27.0%) | 41 (26.5%) |  |  |
| Transverse | 416 (70.6%) | 305 (70.3%) | 111 (71.6%) |  |  |
| Other | 15 (2.55%) | 12 (2.76%) | 3 (1.94%) |  |  |
| **Fascia closure** (Yes): | 543 (90.8%) | 434 (99.1%) | 109 (68.1%) | <0.001 | 598 |
| **Skin closure** (Yes): | 541 (90.5%) | 432 (98.6%) | 109 (68.1%) | <0.001 | 598 |
| **Vacuum treatment** (Yes) | 45 (7.53%) | 36 (8.22%) | 9 (5.62%) | 0.374 | 598 |

**Supplementary Table SM1.** Patients’ characteristics stratified according to clinical outcome (survived versus died) as in the primary publication (1). Data availability is indicated for each variable.

| **Method** | **Domain** | **AUROC** | **AUPRC** | **Brier Score** |
| --- | --- | --- | --- | --- |
| **Logistic Regression** | Demographics | 0.66 (95%-CI: 0.61 - 0.71) | 0.32 (95%-CI: 0.27 - 0.38) | 0.15 (95%-CI: 0.15 - 0.16) |
|  | Physiological | 0.82 (95%-CI: 0.77 - 0.85) | 0.58 (95%-CI: 0.50 - 0.64) | 0.12 (95%-CI: 0.11 - 0.13) |
|  | Surgical | 0.61 (95%-CI: 0.56 - 0.67) | 0.42 (95%-CI: 0.34 - 0.48) | 0.14 (95%-CI: 0.13 - 0.15) |
|  | Multidomain | 0.85 (95%-CI: 0.82 - 0.88) | 0.67 (95%-CI: 0.60 - 0.72) | 0.11 (95%-CI: 0.10 - 0.12) |
| **Elastic Net** | Demographics | 0.66 (95%-CI: 0.61 - 0.70) | 0.31 (95%-CI: 0.27 - 0.37) | 0.15 (95%-CI: 0.15 - 0.16) |
|  | Physiological | 0.82 (95%-CI: 0.77 - 0.85) | 0.57 (95%-CI: 0.49 - 0.64) | 0.12 (95%-CI: 0.11 - 0.13) |
|  | Surgical | 0.60 (95%-CI: 0.55 - 0.66) | 0.41 (95%-CI: 0.33 - 0.47) | 0.14 (95%-CI: 0.13 - 0.15) |
|  | Multidomain | 0.85 (95%-CI: 0.82 - 0.88) | 0.66 (95%-CI: 0.60 - 0.72) | 0.11 (95%-CI: 0.10 - 0.12) |
| **Random Forest** | Demographics | 0.58 (95%-CI: 0.52 - 0.63) | 0.25 (95%-CI: 0.21 - 0.29) | 0.18 (95%-CI: 0.16 - 0.19) |
|  | Physiological | 0.82 (95%-CI: 0.78 - 0.85) | 0.58 (95%-CI: 0.49 - 0.66) | 0.12 (95%-CI: 0.11 - 0.14) |
|  | Surgical | 0.59 (95%-CI: 0.55 - 0.65) | 0.41 (95%-CI: 0.32 - 0.47) | 0.14 (95%-CI: 0.13 - 0.15) |
|  | Multidomain | 0.85 (95%-CI: 0.80 - 0.88) | 0.64 (95%-CI: 0.53 - 0.71) | 0.11 (95%-CI: 0.10 - 0.13) |
| **Gradient Boosting Machine** | Demographics | 0.66 (95%-CI: 0.60 - 0.71) | 0.30 (95%-CI: 0.24 - 0.35) | 0.15 (95%-CI: 0.15 - 0.16) |
|  | Physiological | 0.83 (95%-CI: 0.78 - 0.86) | 0.59 (95%-CI: 0.50 - 0.67) | 0.12 (95%-CI: 0.11 - 0.14) |
|  | Surgical | 0.61 (95%-CI: 0.56 - 0.67) | 0.42 (95%-CI: 0.34 - 0.49) | 0.14 (95%-CI: 0.13 - 0.15) |
|  | Multidomain | 0.85 (95%-CI: 0.81 - 0.89) | 0.65 (95%-CI: 0.56 - 0.73) | 0.11 (95%-CI: 0.10 - 0.13) |
| **Stacked Ensemble** | Demographics | 0.62 (95%-CI: 0.57 - 0.68) | 0.28 (95%-CI: 0.23 - 0.35) | 0.16 (95%-CI: 0.15 - 0.18) |
|  | Physiological | 0.83 (95%-CI: 0.78 - 0.87) | 0.59 (95%-CI: 0.51 - 0.66) | 0.12 (95%-CI: 0.11 - 0.13) |
|  | Surgical | 0.61 (95%-CI: 0.55 - 0.66) | 0.41 (95%-CI: 0.33 - 0.49) | 0.14 (95%-CI: 0.13 - 0.15) |
|  | Multidomain | 0.86 (95%-CI: 0.83 - 0.89) | 0.67 (95%-CI: 0.58 - 0.73) | 0.10 (95%-CI: 0.09 - 0.12) |

AUROC: Area Under the Receiver Operating Characteristic Curve

AUPRC: Area Under the Precision Recall Curve

**Supplementary Table SM2.** Performance metrics for the four base learners and the stacked ensemble. Median and 95%-confidence intervals are shown for the predictions in the training set in random repeated subsampling framework (see Methods).

**Supplementary Figures**


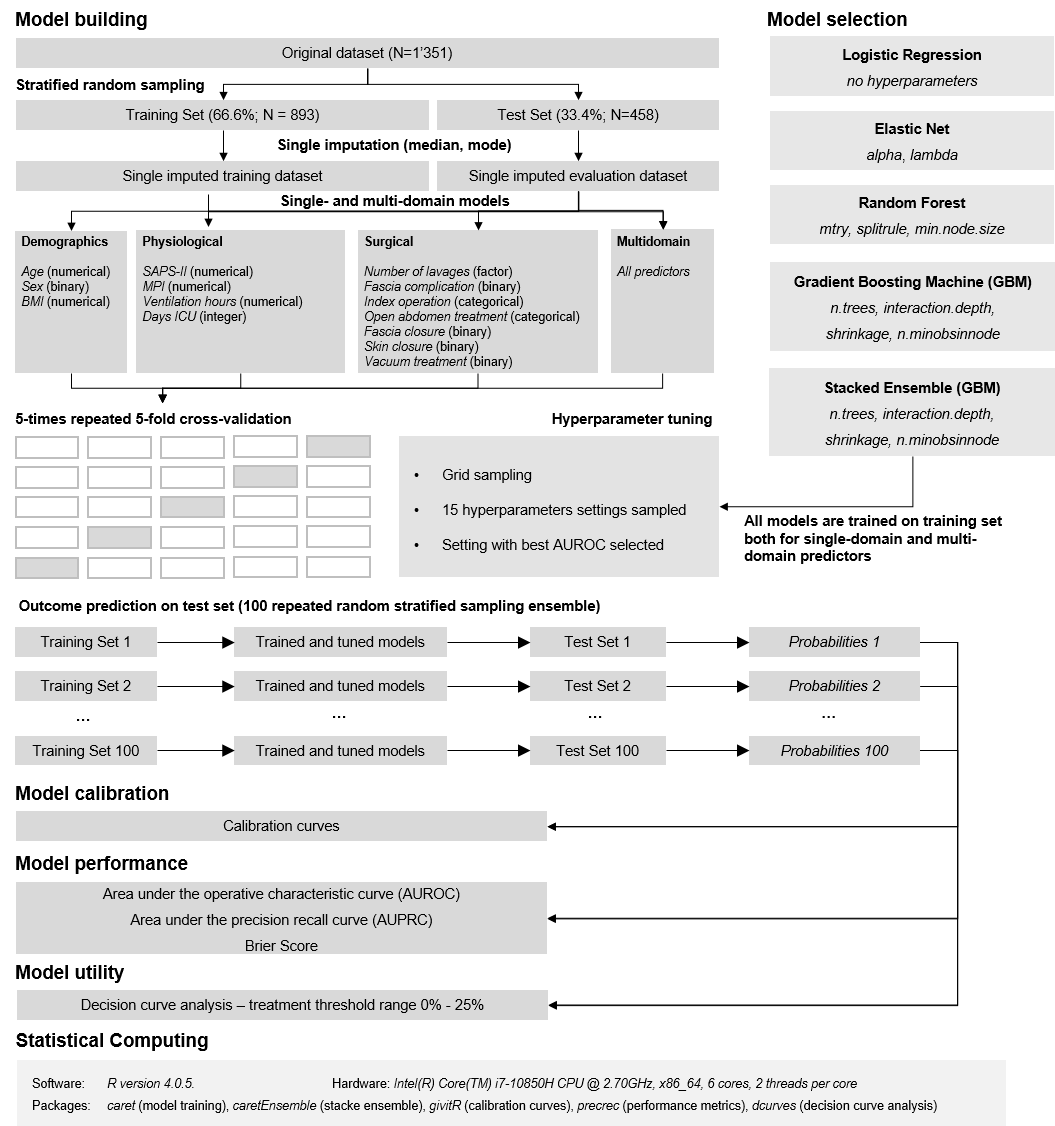


**Supplementary Figure SM1**. Model building and evaluation approach for the entire dataset which contains missing data not at random. A detailed description of the approach is provided in the Methods section.

**
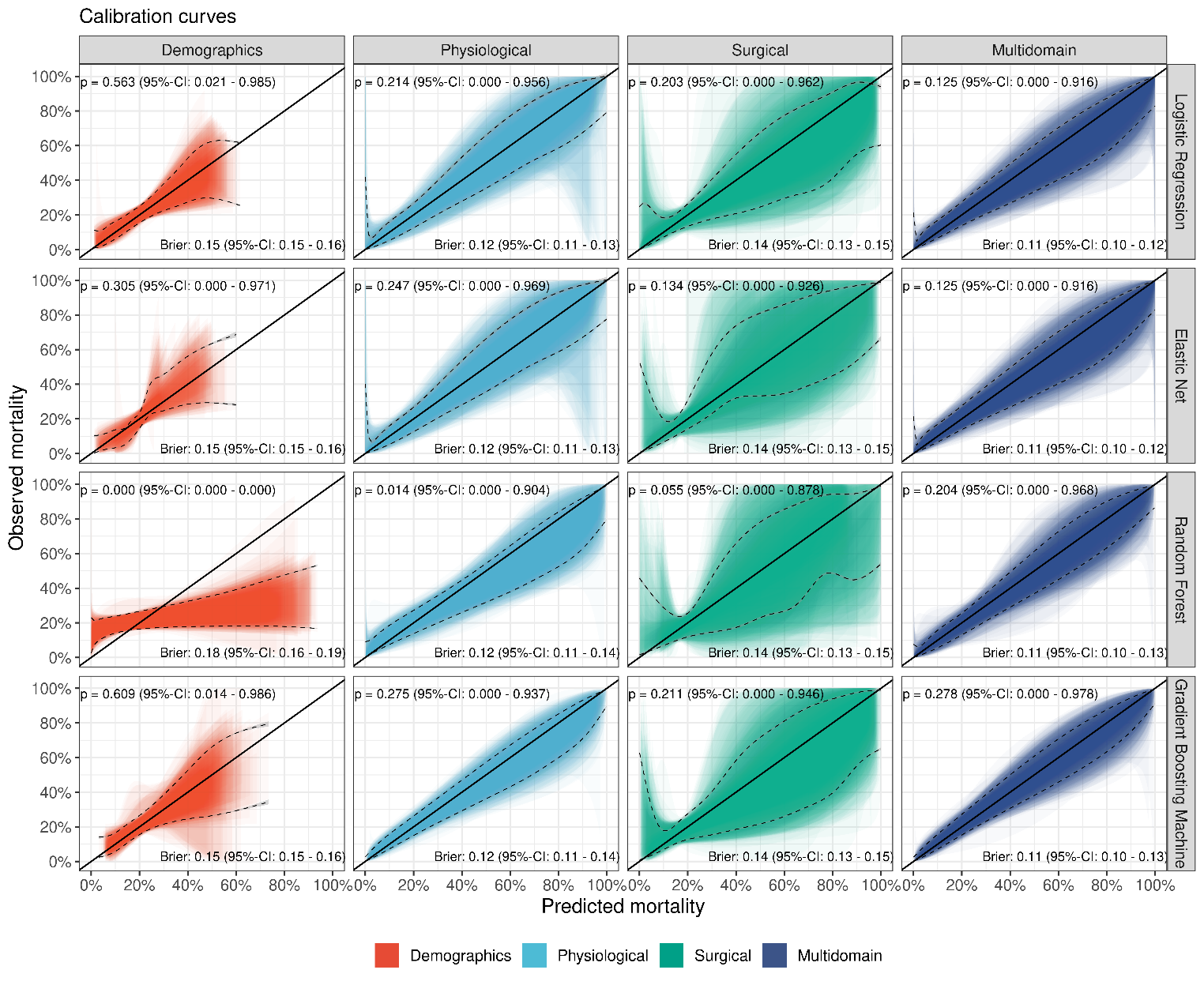
**

**Supplementary Figure SM2**. Calibration curves for the single-domain prediction models and multi-domain predictions models stratified according to the modelling approach based on the entire dataset with single imputation. Shaded areas denote the 95%-confidence intervals. P-values regarding the quality of the calibration (2) and Brier-scores are shown for each prediction model and are summarized by the median and 95%-confidence intervals. Black dashed lines indicate the LOESS-smoothed calibration belts from the ensemble of 100 individual calibration belts.


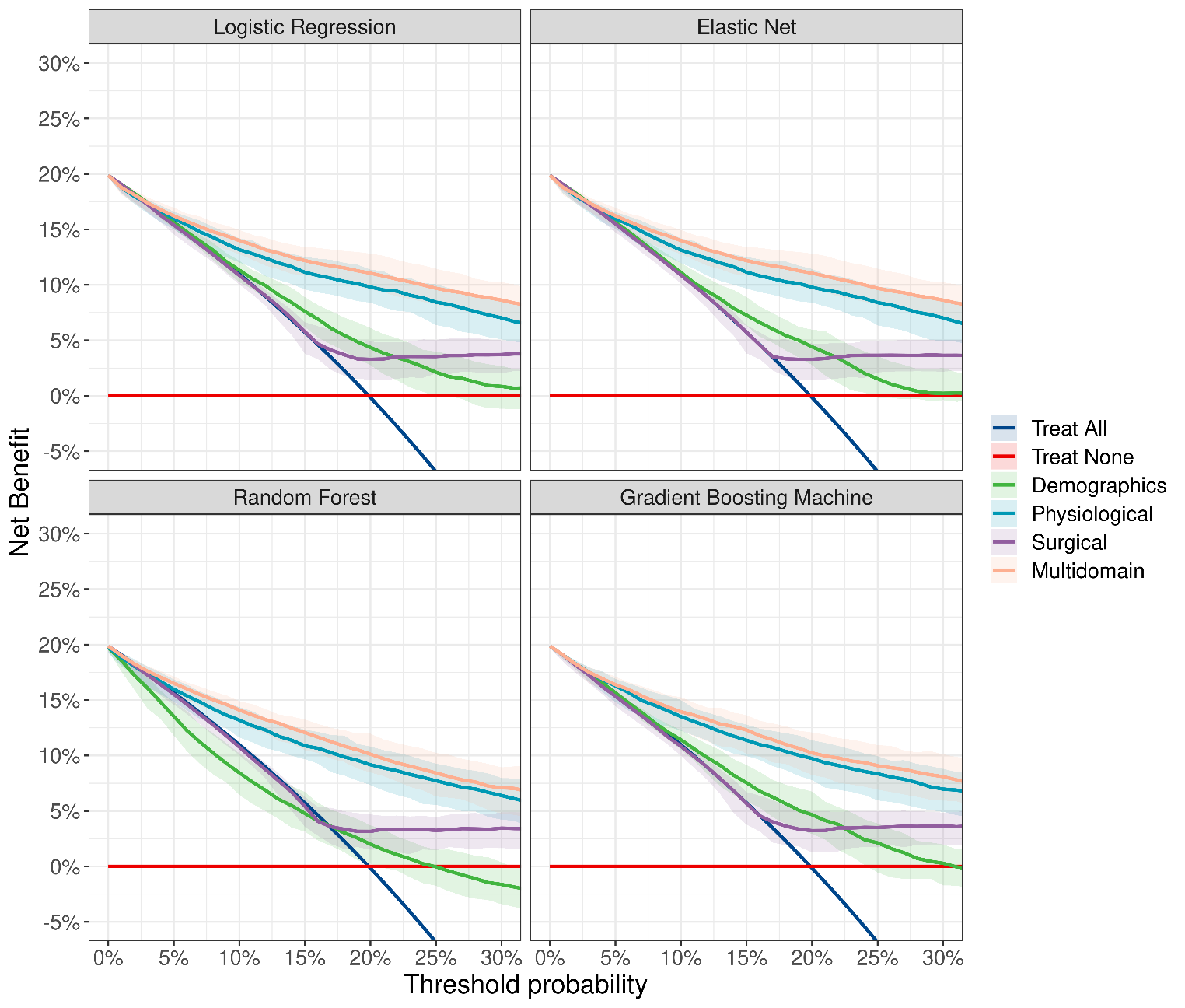


**Supplementary Figure SM3**. Decision curve analysis of single-domain and multi-domain models based on the entire dataset with single imputation. The four domain-specific prediction models are compared to the two default strategies “Treat All” and “Treat None”. Note that the “Treat All” option crosses the zero benefit line at the prevalence of negative outcomes in our cohort including all patients of the original dataset (19.9%).


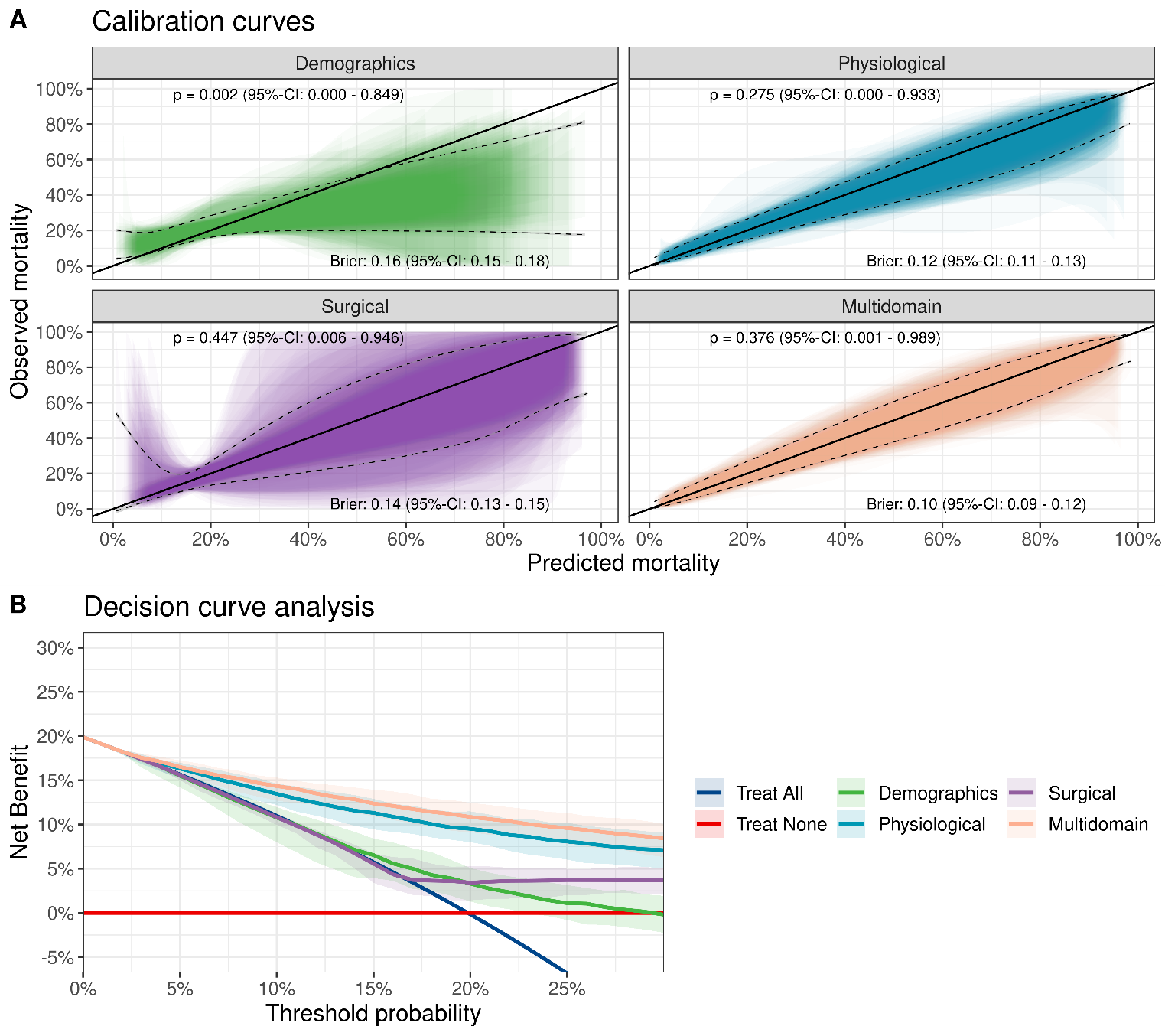


**Supplementary Figure SM4**. Calibration (A) and decision curve analysis (B) for a stacked ensemble prediction model based on a multivariable logistic regression, an Elastic Net, a Random Forest and a Gradient Boosting Machine as base learners based on the entire dataset with single imputation. The stacked ensemble is based on a Gradient Boosting Machine that predicts the mortality outcome based on the cross-validated predictions of the base learners. For calibration, shaded areas denote the 95%-confidence range. P-values regarding the quality of the calibration and Brier-scores are shown.

**References**

1. Petersen S, Huber M, Storni F, Puhl G, Deder A, Prause A, et al. Outcome in patients with open abdomen treatment for peritonitis: a multidomain approach outperforms single domain predictions. Journal of Clinical Monitoring and Computing. 2021.

2. Finazzi S, Poole D, Luciani D, Cogo PE, Bertolini G. Calibration Belt for Quality-of-Care Assessment Based on Dichotomous Outcomes. PLOS ONE. 2011;6(2):e16110.
